# Gut microbiota of sprint athletes: signature microbes and dietary links

**DOI:** 10.3389/fnut.2026.1855417

**Published:** 2026-08-04

**Authors:** Chuanjian Su, Jinfeng Lan, Huimin Chen, Daoming Wang

**Affiliations:** 1 Clinical Medical College of Anhui Medical University, Anhui Medical University, Hefei, Anhui, China; 2 Army Arms University of PLA, Hefei, Anhui, China; 3 The First Affiliated Hospital of Anhui Medical University, Anhui Medical University, Hefei, Anhui, China; 4 School of Physical Education and Health, Anhui Medical University, Hefei, Anhui, China

**Keywords:** college students, diet-microbiota interaction, gut microbiota, metagenomic sequencing, sprint athletes, sprint-associated bacteria

## Abstract

**Background:**

The gut microbiota has emerged as an important biological factor associated with host physiological status in athletes. However, relevant research remains limited for sprint athletes, whose physiological demands differ substantially from those of endurance athletes.

**Objective:**

This study aimed to characterize the gut microbiota profile of college sprint athletes, compare it with non-athletic peers, identify potential sprint-associated bacterial taxa, and explore diet-microbiota associations to propose potential nutritional hypotheses for these signature taxa.

**Methods:**

Fecal samples were collected from 20 college sprint athletes and 23 non-athletic college students for metagenomic sequencing. Dietary intake was assessed using a validated food frequency questionnaire. Alpha and beta diversity analyses were performed to evaluate microbial community diversity and structure. LEfSe was used to identify differentially abundant taxa. Functional annotation and enrichment were conducted using KEGG, GO, and other databases. Spearman’s correlation was applied to examine diet-microbiota relationships.

**Results:**

Alpha diversity indices (Shannon, Chao1, etc.) did not differ significantly between groups. In contrast, beta diversity analysis revealed significant structural separation. LEfSe identified *Segatella copri* (LDA = 4.986, *p* = 0.017) and *Bifidobacterium adolescentis* (LDA = 3.154, *p* = 0.003) as signature taxa in athletes, both with significantly higher abundance than in non-athletes. Functional analysis showed predicted enrichment of pathways related to energy metabolism (carbohydrate metabolism, ATP binding) and amino acid metabolism in athletes. Correlation analysis indicated that *S. copri* abundance was nominally positively associated with dairy intake (*r* = 0.31, *p* = 0.045), while *B. adolescentis* was nominally associated with whole grains, soy milk/soy powder, and dairy products.

**Conclusion:**

College sprint athletes possess a distinct gut microbiota structure compared with non-athletes. *S. copri* and *B. adolescentis* represent distinct microbial signatures associated with sprint athletes, accompanied by corresponding predicted functional pathway enrichment. Specific dietary patterns, including the intake of whole grains, soy milk/soy powder, and dairy products, exhibit exploratory nominal associations with the signature gut microbiota composition of sprinters.

## 1 Introduction

Sprinting is an anaerobic-dominant sport that requires explosive power, rapid energy supply, and efficient metabolic regulation (1, 2). Recent studies have highlighted the gut microbiota as an important biological factor associated with host physiological status and metabolic characteristics in athletes, particularly in relation to energy metabolism, nutrient absorption, inflammation regulation, and lactate clearance (3–5). The majority of relevant studies have centered on endurance athletes (e.g., marathon runners, cyclists) (6–8). Although emerging research has profiled the microbiome and its functions in strength and combat athletes, including weightlifters and wrestlers, few investigations have focused on track sprinters (9–11). High-throughput metagenomic data focusing specifically on track sprinters who possess distinct anaerobic metabolic demands remain unavailable.

Differences in training modes (anaerobic vs. aerobic) lead to distinct physiological adaptations, which may induce corresponding changes in gut microbiota composition (12, 13). For example, endurance athletes often show an increased abundance of lactate-utilizing bacteria (e.g., *Veillonella*), which enhance exercise runtime by metabolizing exercise-induced lactate into propionate (14). Identifying the gut microbiota characteristics of sprint athletes and their potential exercise-associated taxa is essential for developing targeted interventions.

Diet is a primary determinant of gut microbiota structure, and dietary optimization to regulate the microbiome is increasingly recognized as a promising approach in sports nutrition to support physiological adaptation (15, 16). However, diet-microbiota interactions specific to sprint athletes remain unclear. Clarifying how dietary components (e.g., carbohydrate type, protein intake, fruit and vegetable consumption) correlate with key microbial taxa can provide hypothesis-generating evidence for future controlled dietary studies.

Using metagenomic sequencing, this study comprehensively profiled the gut microbiota of college sprint athletes, compared it with non-athletic controls, identified candidate sprint-associated bacteria, and explored nominal diet-microbiota associations. Our findings aim to fill the knowledge gap regarding sprint athletes’ gut microbiota and provide preliminary, hypothesis-generating insights into potential exploratory dietary links within this specialized cohort.

## 2 Materials and methods

### 2.1 Study participants

Twenty sprint college athletes (Athletes group) were recruited from the track and field team of Anhui Medical University, with regular high-intensity sprint training and no history of metabolic or gastrointestinal diseases. Twenty-three non-athletic college students (non-athletic group) were recruited from general college courses, with no regular physical training and matching age and BMI with the athlete group. All participants provided written informed consent, and the study was approved by the Ethics Committee of Anhui Medical University.

### 2.2 Physical activity data collection

Physical activity was quantified using the FITT framework (17). To eliminate inter-device variability, all participants wore identical smart wristbands (Mi Band 10, Xiaomi Inc., Beijing, China) to record continuous heart rate (HR) via photoplethysmography (PPG). Raw logs were filtered to remove motion artifacts (HR outside 30–230 bpm or sudden changes >15 bpm) and incomplete sessions (signal dropouts >5 min or tracking duration <85%). Subjective exercise intensity was assessed within 30 min post-exercise using the Borg CR10 Scale, yielding scores of 8–10 for athletes and 4–6 for non-athletes. The entire monitoring period lasted for 1 week with daily data logging, culminating in the collection of fecal samples and the completion of dietary questionnaires at the end of the tracking window.

### 2.3 Sample collection and dietary survey

Fecal samples (≥5 g) were collected using sterile sampling tubes and stored at −80 °C within 2 h. We assessed dietary information with a semiquantitative food-frequency questionnaire (FFQ). The FFQ utilized in our study was revised based on the validated FFQ created by the Chinese Center for Disease Control and Prevention, recording the weekly intake of 24 food categories (18) including grains, vegetables, fruits, proteins, and processed foods. The structural details of these questionnaires are provided in Supplementary Data 1. The questionnaire was administered during the exact week of fecal sample collection to capture synchronous dietary information, and the total intake for this week was recorded as the weekly intake volume.

### 2.4 Metagenomic sequencing and bioinformatics analysis

Fecal DNA was extracted using the FastDNA Spin Soil Kit (MP Biomedicals, United States) following the manufacturer’s protocol. DNA quality was assessed using Agilent 5,400 and Qubit 4.0. Metagenomic libraries were constructed with insert sizes of 300–350 bp and sequenced on the Illumina NovaSeq Xplus platform (PE150).

Raw reads were filtered using fastp (v0.23.4) (19) to remove adapters and low-quality sequences. Host contamination was eliminated using Bowtie2 (v2.3.5.1) (20). Clean reads were assembled with MEGAHIT (v1.2.9) (21), and CDS prediction was performed using MetaGeneMark (v3.38) (22). Non-redundant Unigene sets were obtained using MMseqs2 (23). Taxonomic annotation was conducted using Diamond (v2.1.9) (24) against the NR database. Functional annotation was performed based on KEGG, GO, eggNOG, and CAZy databases.

Alpha diversity indices (Shannon, Chao1, Simpson, etc.) were calculated using QIIME2 (v2024.02) (25). Beta diversity was analyzed through PCoA based on Bray-Curtis. Adonis analyses were used to test group differences. Differentially abundant taxa were identified using LEfSe (26), with a strict selection threshold set at an LDA score > 3.0 and the multi-test significance controlled at a false discovery rate (FDR) < 0.2. To account for the compositional nature of the microbial abundance data and validate the robustness of the identified signatures, an alternative association analysis was performed. Zero counts in the microbial profiles were replaced with a pseudocount equal to 0.5 times the minimum non-zero value, and a centered log-ratio (CLR) transformation was applied to the count abundance of each feature. Subsequently, linear regression models were constructed to examine the robust associations between individual CLR-transformed taxa and groups. Functional enrichment analyses were performed using clusterProfiler (27) and ReporterScore (28). Correlation analysis between diet and microbiota was conducted using Spearman’s correlation coefficient.

### 2.5 Statistical analysis

Statistical analysis was performed using R (v4.3.1) and SPSS (v26.0). Continuous variables were presented as mean ± SD, and group comparisons were conducted using independent samples *t*-test or Mann–Whitney U test. Categorical variables were compared using chi-square test. Correlations between dietary factors and microbiota were analyzed using Spearman’s correlation, with unadjusted nominal *p* < 0.05 considered significant for exploratory screenings. FDR < 0.05 was considered statistically significant. For microbial differential analysis via LEfSe, significance was defined at an FDR < 0.2 to balance biomarker discovery.

## 3 Results

### 3.1 Study population characteristics

The athletes group (20 participants: 16 males, 4 females) had a mean age of 20.3 ± 1.2 years and BMI of 22.4 ± 1.8 kg/m^2^. The non-athletes group (23 participants: 18 males, 5 females) had a mean age of 20.1 ± 1.3 years and BMI of 22.1 ± 2.0 kg/m^2^. No significant between-group differences were observed in age, sex, or BMI (*p* > 0.05), ensuring that baseline demographics did not confound gut microbiota comparisons (Table 1).

**Table 1:** Study population characteristics.

Characteristics	Athletes (M1)	Non-athletes (M2)
Baseline		
Age (year)s	20.3 ± 1.2	20.1 ± 1.3
BMI (kg/m^2^)	22.4 ± 1.8	22.1 ± 2.0
Gender	16 males, 4 females	18 males, 5 females
FITT^a^/week		
Frequency^b^	5.0 ± 0.5 sessions/week	2.5 ± 1.0 sessions/week
Intensity^c^	85–95% HRmax, RPE 8–10	60–75% HRmax, RPE 4–6
Time^d^	10.3 ± 1.4 h/week (120–150 min/session)	2.2 ± 1.6 h/week (30–60 min/session)
Type^e^	Sprint & explosive strength training	Recreational aerobic & ball sports

a FITT is the core framework of exercise prescription in exercise science, with the full English names as follows: Frequency, Intensity, Time, Type.

b The number of exercise/training sessions per week.

c The load or exertion level of exercise, quantified by maximum heart rate (HRmax), Rating of Perceived Exertion (RPE). Data were collected from sports wristbands.

d The duration of a single exercise session (including warm-up, sprint drills, strength training, cool-down), or total weekly exercise time. Data were collected from sports wristbands.

e The specific form or category of exercise, Athletes: 100 m/200 m/400 m sprints, starting block drills, hurdle drills Auxiliary training: explosive strength training (squat, deadlift, depth jump), speed endurance interval running, core training, flexibility training. Non-athletes: jogging, brisk walking, campus aerobics, badminton, basketball, swimming, etc. Mainly aerobic leisure exercise without specialized training.

Exercise habits were evaluated using the FITT principle (Frequency, Intensity, Time, Type) (17) (Table 1). The athletes group trained 5.0 ± 0.5 sessions per week at 85–95% HRmax with an RPE of 8–10, representing high-intensity anaerobic effort. Weekly training time totaled 10.3 ± 1.4 h, with sessions lasting 120–150 min (warm-up, sprint drills, strength training, cool-down). Training focused on sprints (100 m/200 m/400 m) and explosive strength (squats, deadlifts, depth jumps, speed endurance intervals, core work, flexibility). The non-athletes group exercised 2.5 ± 1.0 times weekly at 60–75% HRmax (RPE 4–6), with total weekly activity of 2.2 ± 1.6 h and sessions of 30–60 min. Activities were recreational aerobic and ball sports (jogging, walking, aerobics, badminton, basketball, swimming) without specialized sprint or strength training. All FITT data were recorded via sports wristbands.

### 3.2 Dietary questionnaire results

Dietary intake across 24 categories was assessed in 20 sprint athletes (athletes) and 23 non-athletic students (non-athletes) (Table 2; Supplementary Data 1). Independent-samples *t*-tests showed that athletes consumed significantly more dairy products and baked goods per week than non-athletes (both *p* < 0.05). No significant differences were detected in the remaining 22 food items. These results define the distinct nutritional baseline patterns characteristic of each study cohort.

**Table 2:** Dietary questionnaire results.

Dietary Item	Athletes (M1) Mean ± SD (g)	Non-athletes (M2) Mean ± SD (g)	*p*
Rice	1207.5 ± 539.4	930.4 ± 476.5	0.136
Steamed wheat flour products	401.3 ± 481.6	289.1 ± 245.8	0.345
Whole grains	139.1 ± 152.3	54.2 ± 79.6	0.114
Tubers	130.6 ± 137.5	89.7 ± 106.2	0.407
Fried pastries	58.8 ± 86.1	34.8 ± 38.9	0.573
Soy products	113.8 ± 106.7	105.1 ± 63.2	0.56
Soy milk & soy powder	206.3 ± 249.8	162.8 ± 218.5	0.478
Mushrooms & algae	78.8 ± 114.2	53.9 ± 58.8	0.382
Fresh vegetables	481.3 ± 347.6	449.6 ± 388.7	0.682
Fresh fruits	320.0 ± 395.5	137.5 ± 154.3	0.141
Citrus fruits	65.0 ± 113.2	26.8 ± 39.8	0.115
Dried fruits	36.3 ± 59.4	59.2 ± 136.5	0.571
Nuts	85.0 ± 135.7	56.8 ± 128.6	0.583
Animal liver	36.3 ± 73.5	32.2 ± 66.9	0.778
Animal blood	17.5 ± 20.6	20.1 ± 26.9	0.834
Shrimp	31.3 ± 40.5	41.2 ± 126.8	0.752
Fish	40.0 ± 52.1	32.8 ± 40.5	0.498
Mollusks	8.8 ± 13.1	14.4 ± 22.5	0.537
Shellfish	12.5 ± 22.4	17.2 ± 28.6	0.684
Eggs	345.0 ± 385.6	376.8 ± 331.9	0.678
Dairy products	641.3 ± 768.2	216.5 ± 310.2	0.044*
Baked goods	328.8 ± 415.3	106.9 ± 89.7	0.041*
Sugary snacks	51.3 ± 85.7	50.2 ± 53.8	0.955
Puffed snacks	45.0 ± 82.4	102.8 ± 151.7	0.176

* *p* < 0.05.

### 3.3 Gut microbiota diversity and structure

**Figure 1:**
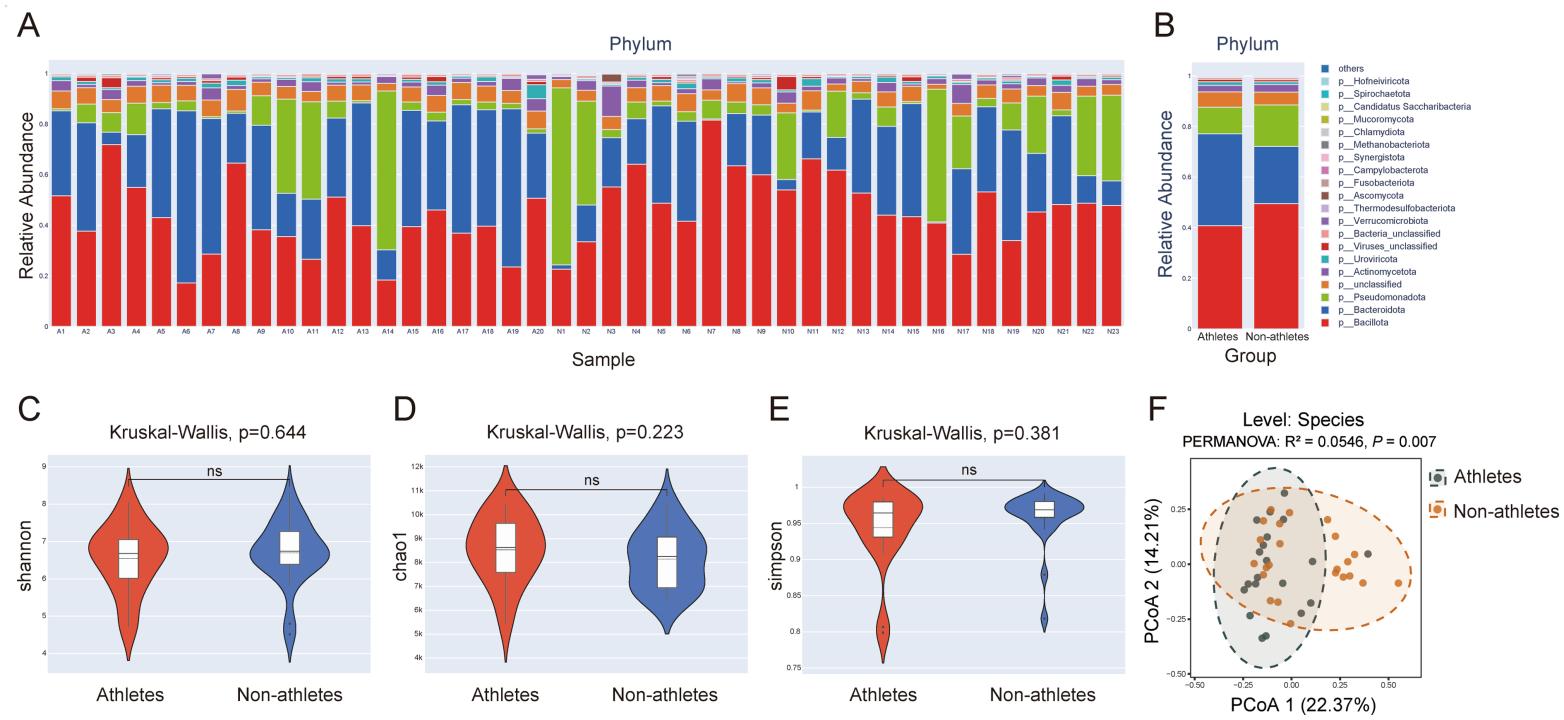
Gut microbiota composition, alpha diversity, and beta diversity between sprint athletes (athletes) and non-athletes (non-athletes). **(A)** Phylum-level gut microbiota composition in each individual sample. **(B)** Average phylum-level gut microbiota composition in athletes and non-athletes groups. **(C–E)** Comparisons of alpha diversity indices including Shannon **(C)**, Chao1 **(D)**, and Simpson **(E)** between the two groups. ns, not significant. **(F)** Principal coordinate analysis (PCoA) plot of gut microbial community structure at the species level based on Bray-Curtis distance (*R*^2^ = 0.0546, *p* = 0.007, PERMANOVA). Ellipses represent 95% confidence intervals for athletes and non-athletes.

The gut microbiota composition at the phylum level was analyzed across all samples (Figure 1A; Supplementary Data 2). The dominant phyla in both groups were *Firmicutes* (*Bacillota*), *Bacteroidota*, and *Pseudomonadota*, collectively accounting for over 85% of the total microbial abundance in all individuals. As shown in the group-averaged composition, athletes exhibited a distinct phylum-level profile compared to non-athletes. Specifically, athletes had a higher relative abundance of Bacteroidota (36.3% vs. 22.6% in non-athletes), while non-athletes showed a slightly higher proportion of Firmicutes (49.5% vs. 40.8% in athletes) (Figure 1B). These differences indicate that regular sprint training is associated with a distinct profile in the dominant phyla of the gut microbiota, characterized by an increased *Bacteroidota/Firmicutes* ratio in athletes.

To evaluate the overall characteristics of gut microbial communities, alpha diversity indices were first analyzed, including Shannon (species diversity), Chao1 (species richness), Simpson (dominance). The results showed that there were no significant differences in any of these indices between the athletes and non-athletes groups (Figures 1C–E). Additionally, dilution curves for all alpha diversity indices tended to flatten, indicating that the sequencing depth was sufficient to capture the majority of microbial species in the samples, and no further sequencing would significantly increase the number of observed species (Supplementary Figures S1A–C).

While alpha diversity did not differ between groups, beta diversity analysis revealed distinct differences in microbial community structure. PCoA analysis based on Bray-Curtis distance showed that samples from the athletes and non-athletes groups formed relatively separate clusters, with PCoA1 and PCoA2 accounting for 22.37 and 14.21% of the total variance, respectively, and samples from the two groups showed clear separation (Figure 1F). This structural separation was statistically significant as evaluated by PERMANOVA (*R*^2^ = 0.0546, *p* = 0.007). Multivariate homogeneity of dispersions analysis further confirmed that the beta dispersion within each group was highly comparable (Supplementary Figure S1D). NMDS analysis further confirmed this pattern, with a stress value of 0.18 (less than 0.2), indicating reliable results and distinct clustering of the two groups (Supplementary Figure S1E). These results collectively indicate that although sprint athletes and non-athletes have similar levels of gut microbial diversity, sprint athletes exhibit a distinct gut microbiota community structure compared to non-athletes.

### 3.4 Differentially abundant taxa

**Figure 2:**
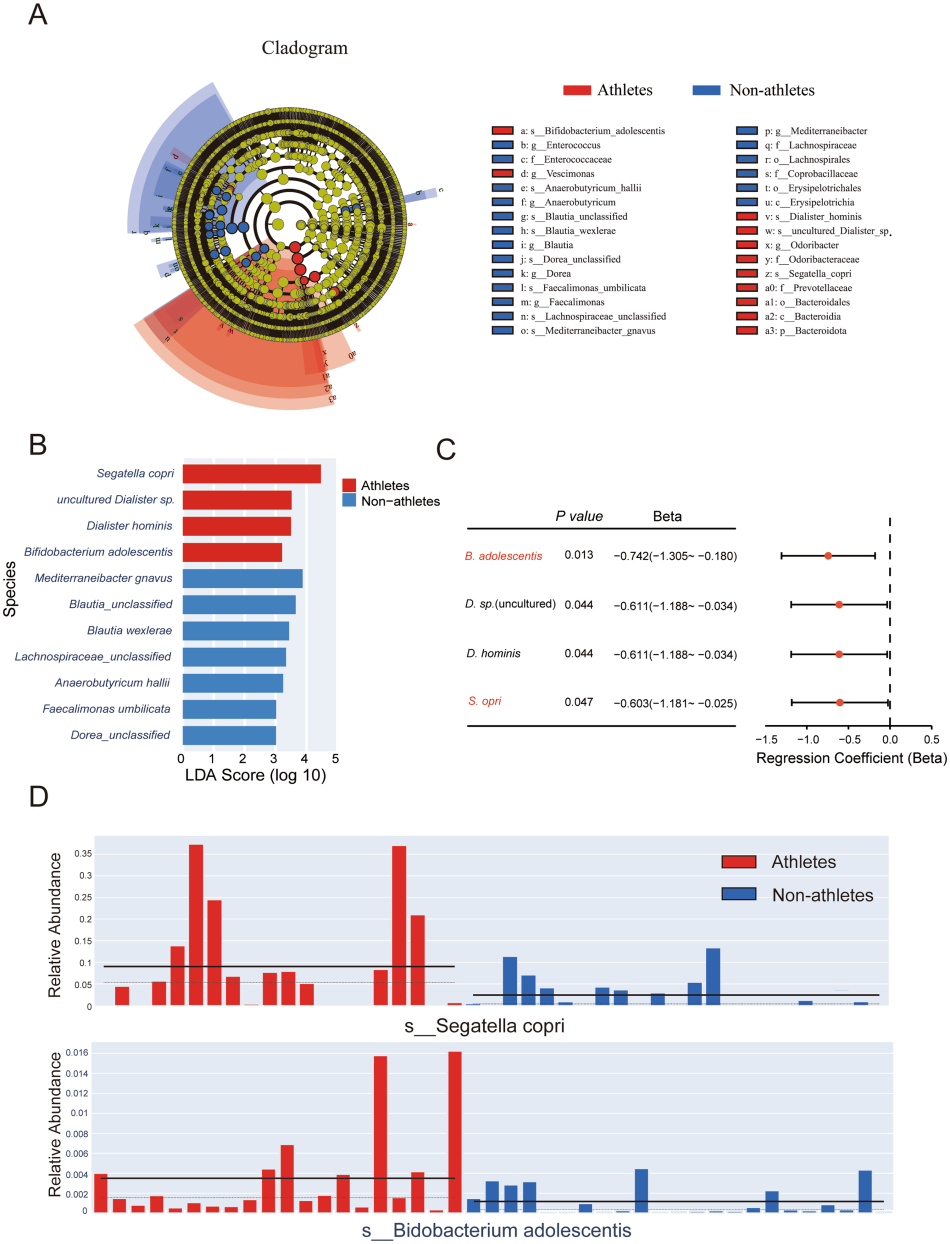
Differential gut microbial taxa between sprint athletes and non-athletes. **(A)** LEfSe analysis identified differentially abundant taxa with LDA score >3.0 and FDR < 0.2. **(B)** LDA scores of differentially abundant taxa in the athletes (red) and non-athletes (blue) groups. **(C)** Forest plot showcasing the robust associations between CLR-transformed microbial taxa and groups based on linear regression analyses, displaying the corresponding regression coefficients (Beta) and 95% confidence intervals. **(D)** Bar plots illustrating the relative abundance distribution of the two core signature bacteria, *S. copri* and *B. adolescentis*, across all individual samples in both groups.

To identify specific microbial taxa contributing to the structural differences, multiple statistical methods were employed. LEfSe analysis (LDA > 3, FDR < 0.2) identified 30 differentially abundant taxa between the Athletes and Non-athletes groups, with 11 taxa significantly enriched in the Athletes group and 19 enriched in the non-athletes group (Figure 2A). Among these, *Segatella copri* (phylum Bacteroidota, class Bacteroidia, order Bacteroidales, family Prevotellaceae, genus Segatella) was the most significant signature taxon of the athletes group, with an LDA score of 4.986 and a *p*-value of 0.017 (Figure 2B; Supplementary Data 3). Notably, *Bifidobacterium adolescentis* was significantly enriched in the athletes group (LDA score = 3.154, *p* = 0.003). As a well-recognized probiotic, *B. adolescentis* has been documented to exhibit functional changes reported in the context of high-intensity exercise-related studies (29), which aligns with the predicted functional potential observed in our sprint cohort (Figure 2B; Supplementary Data 3). To account for the compositional nature of the metagenomic data, we further applied a centered log-ratio (CLR) transformation followed by linear regression analysis to validate these findings. This independent analysis demonstrated that the key bacterial signatures identified in the athletes group remained highly consistent (Figure 2C). Quantitative analysis showed that the average abundance of *S. copri* and *B. adolescentis* in the athletes group was significantly higher than that in the non-athletes group (Figure 2D).

In addition to *S. copri* and *B. adolescentis*, other taxa enriched in the athletes group included *Dialister hominis*. In contrast, the non-athletes group exhibited significantly higher abundance of several bacterial taxa with context-dependent or potentially pro-inflammatory properties, including *Mediterraneibacter gnavus*, *Blautia wexlerae*, *Anaerobutyricum hallii*, and *Faecalimonas umbilicata* (Supplementary Data 3).

### 3.5 Functional characteristics of gut microbiota

**Figure 3:**
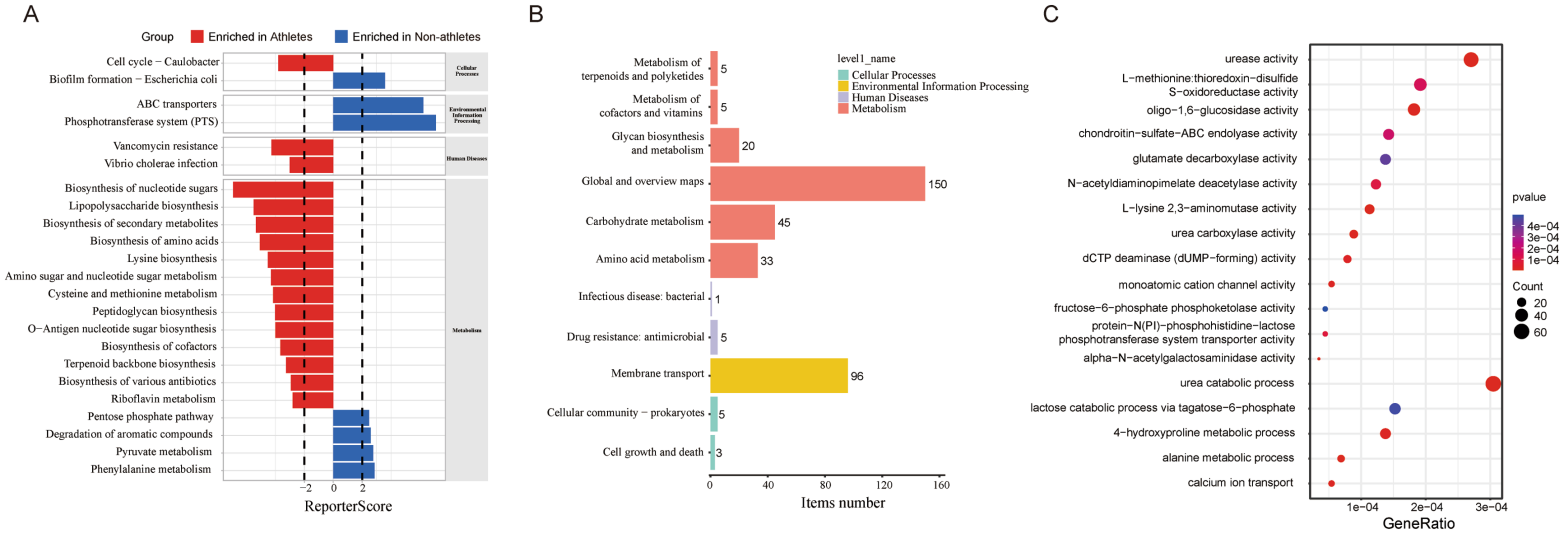
Functional characteristics of gut microbiota in sprint athletes and non-athletes. **(A)** Bar plot of enriched KEGG pathways with |Reporter score| > 2. Red and blue bars represent significant enrichment in the athletes and non-athletes groups, respectively. **(B)** Bar plot illustrating the distribution of differentially abundant KEGG Orthologies (KOs) at KEGG level 2. The length of each bar represents the total number of significantly altered items within each pathway, classified and colored according to their respective KEGG level 1 functional categories. **(C)** Dot plot of significantly enriched GO terms. The *x*-axis indicates the GeneRatio, while the dot size represents the count of enriched items. The color gradient reflects the statistical significance based on the *p*-value.

To explore the functional implications of the observed taxonomic differences, functional annotation and enrichment analyses were performed based on multiple databases. Specifically, based on the KEGG database, the gut microbiota of the athletes was predominantly enriched in pathways related to carbohydrate metabolism and amino acid metabolism such as biosynthesis of nucleotide sugars, Biosynthesis of amino acids, Lysine biosynthesis, and Amino sugar and nucleotide sugar metabolism (Figure 3A; Reporter Score >2, FDR < 0.05). Consistently, at KEGG level 2, Carbohydrate metabolism and Amino acid metabolism harbored high numbers of pathways within the metabolic category, containing 45 and 33 items, respectively (Figure 3B; Supplementary Data 4). These enrichments indicated that the gut microbiota of sprint athletes showed enhanced functional potential for carbohydrate breakdown and amino acid metabolism.

GO analysis further supplemented the functional characteristics of the gut microbiota in athletes, as shown in Figure 3C. The results indicated that athletes were mainly enriched in GO terms related to urease activity and amino acid metabolism (FDR < 0.05). Urease activity plays a role in nitrogen metabolism, helping to regulate the intestinal environment and support nutrient utilization, while the enrichment of amino acid metabolism-related GO terms further suggested a higher functional potential of athletes’ gut microbiota to synthesize and utilize amino acids. Concurrently, these findings align with the KEGG pathway profiles, collectively suggesting that the gut microbiota of sprint athletes exhibits a distinct predicted functional profile characterized by distinct carbohydrate and amino acid metabolic potentials.

### 3.6 Diet-microbiota associations

**Figure 4:**
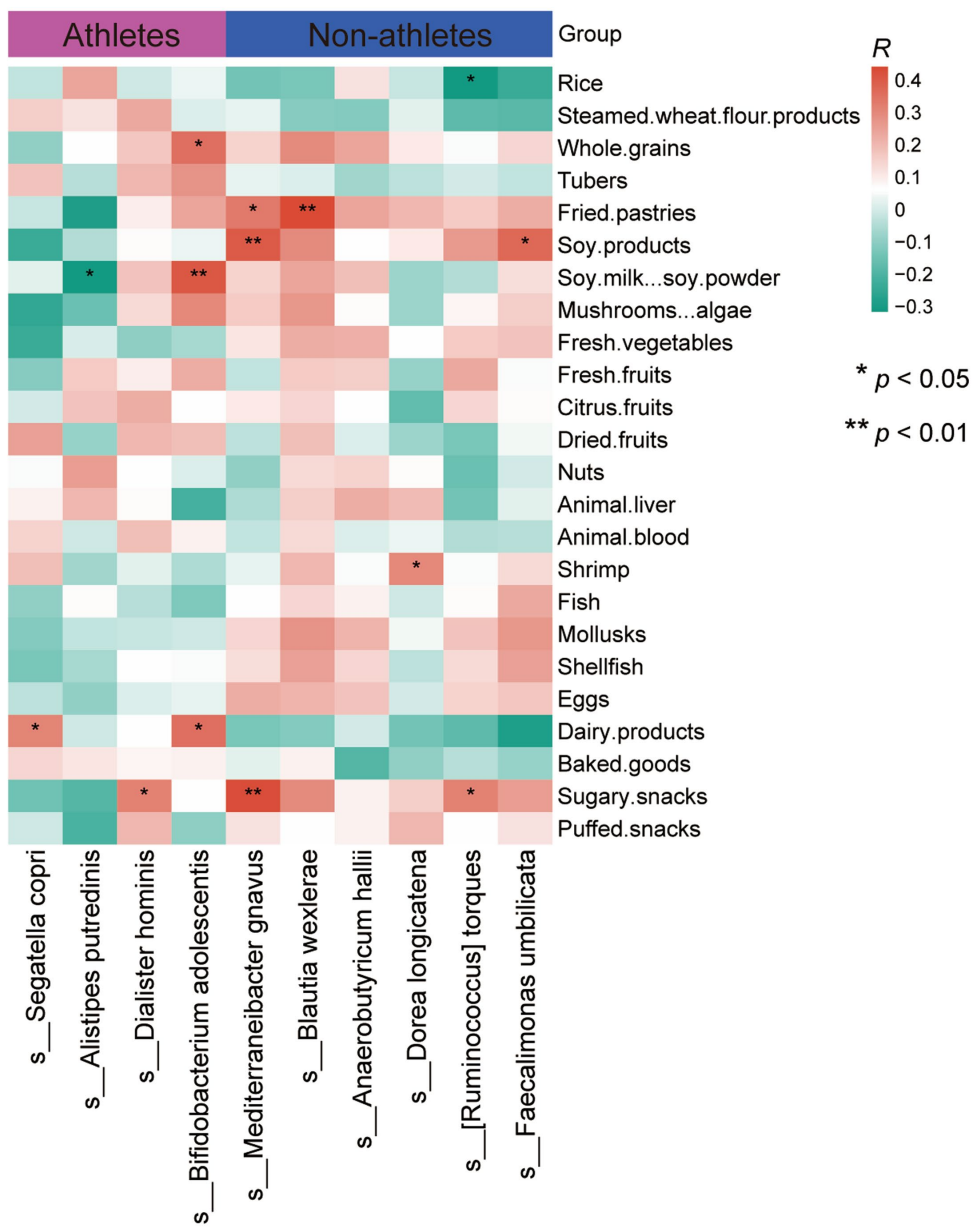
Dietary intake correlates with gut microbial composition in college sprint athletes and non-athletes. **(A)** Spearman’s correlation analysis the relationship between dietary factors and the identified key microbial taxa. * *p* < 0.05, ** *p* < 0.01 (unadjusted).

To investigate the relationship between dietary factors and the identified key microbial taxa, Spearman’s correlation analysis was performed. The results showed that the abundance of *S. copri*, the top-ranking signature taxon of sprint athletes, exhibited a nominal positive correlation with the intake of dairy products (*r* = 0.31, *p* = 0.045) (Figure 4A).

Concurrently, *B. adolescentis* exhibited nominal positive correlations with the intake of whole grains, soy milk & soy powder, and dairy products (Figure 4A). These findings highlight speculative co-variations between specific dietary components and sprint-associated bacteria. In addition, the bacterial taxa enriched in the non-athletes group were nominally associated with soy products, fried pastries, and sugary snacks (Figure 4A). These exploratory screening results indicate that distinct dietary habits align with the respective gut microbiota structures observed in sprint athletes and non-athletes.

## 4 Discussion

This study systematically characterized the gut microbiota of sprint college athletes, identified key differences from non-athletes, and explored preliminary diet-microbiota associations. The main findings are: (1) Sprint athletes and non-athletes have similar microbial diversity but distinct community structures; (2) *S. copri* and *B. adolescentis* represent signature taxa highly enriched in sprint athletes; (3) Functional pathways related to energy metabolism and nutrient transport are enriched in athletes’ gut microbiota; and (4) Specific dietary patterns, such as the intake of whole grains, soy milk/soy powder, and dairy products, exhibit exploratory, nominal associations with these identified key microbial signature taxa.

### 4.1 Gut microbiota diversity and structure differences

Unlike some studies on endurance athletes showing increased alpha diversity (3, 30–32), our results found no significant differences in alpha diversity between sprint athletes and non-athletes. This may be due to the different physiological demands of sprinting (anaerobic, short-duration) versus endurance sports (aerobic, long-duration). Endurance training induces more profound changes in gut barrier function and nutrient metabolism, leading to increased microbial diversity (33), while sprint training may primarily alter microbial community structure rather than diversity.

The significant difference in beta diversity confirms that athlete status was significantly associated with alterations in gut microbiota structure, consistent with previous studies showing that exercise type affects microbial community composition (34–37). The distinct clustering of microbiota between athletes and non-athletes suggests that regular sprint training is associated with a distinct microbial community structure, reflecting preliminary predicted functional signatures that align with the metabolic characteristics of anaerobic exercise.

### 4.2 Potential sprint-associated bacteria

*S. copri* was identified as the top-ranking signature taxon of sprint athletes, with significantly higher abundance in athletes. *S. copri* belongs to the family *Prevotellaceae* and has been reported to be associated with carbohydrate metabolism and short-chain fatty acid (SCFA) production (38–40). SCFAs (e.g., propionate, butyrate) are important energy sources for intestinal epithelial cells and can enhance glucose metabolism and insulin sensitivity (41, 42). These pathways offer a tentative correlational rationale linking the metabolic potential of *S. copri* to host glucose substrate availability.

*B. adolescentis*, a well-documented probiotic, was also significantly enriched in sprint athletes and may serve as another notable sprint-associated bacterium. As a core member of Bifidobacterium, *B. adolescentis* has been reported to sustain intestinal mucosal barrier integrity, suppress colonization of opportunistic pathogens, and relieve intestinal inflammation and oxidative stress observed in association with high-intensity exercise-related conditions (43, 44). Meanwhile, *B. adolescentis* participates in the fermentation of dietary fiber and resistant starch to produce acetate and other short-chain fatty acids that serve as energy substrates in glycolipid metabolism (45). Furthermore, *B. adolescentis* has been documented to promote the absorption and utilization of amino acids, calcium and other nutrients (46, 47). These documented physiological traits provide a reasonable hypothesis-generating interpretation for the distinct microbial profile observed in sprinters.

### 4.3 Limitations

This study has several limitations. First, the sample size is relatively small which may reduce statistical robustness and increase the likelihood of chance findings due to individual microbiome variability, and larger cohorts are needed to confirm the findings. Second, dietary assessment via self-reported questionnaires may introduce recall bias and limit quantitative precision. Consequently, potential confounders including total energy intake, supplement use, and training load variations were not fully controlled. Third, the causal relationship between microbiota and sprint performance was not established; intervention studies (e.g., probiotic supplementation, dietary intervention) are needed to clarify whether these signature bacteria directly influence sprint performance. Finally, confounding factors such as sleep quality, stress levels, and supplement use were not fully controlled, which may influence gut microbiota composition.

## 5 Conclusion

Sprint college athletes have distinct gut microbiota structures compared to non-athletic peers, with *S. copri* and *B. adolescentis* as key signature bacterial taxa. Specific dietary patterns, including the intake of whole grains, soy milk/soy powder, and dairy products, exhibit exploratory, nominal associations with the signature gut microbiota composition of sprinters. This study provides new insights into the gut microbiota of sprint athletes, offering hypothesis-generating evidence for future controlled dietary studies based on microbiome links.

## Data Availability

The datasets presented in this study can be found in online repositories. The names of the repository/repositories and accession number(s) can be found in the article/Supplementary material.

## Supplementary material

SUPPLEMENTARY FIGURE S1 Rarefaction curves and beta diversity analyses of gut microbiota. **(A–C)** Rarefaction curves for Shannon, Chao1, and observed species indices, indicating sufficient sequencing depth. **(D)** Boxplot of multivariate homogeneity of groups dispersions (beta dispersion) based on Bray-Curtis distance. **(E)** Non-metric multidimensional scaling (NMDS) plot (stress = 0.18) confirming distinct microbial community structure between groups.

## References

[1] Briand J di Prampero PE Osgnach C Thibault G Tremblay J. Quantifying metabolic energy contributions in sprint running: a novel bioenergetic model. Eur J Appl Physiol. (2025) 125:3521–41. doi: 10.1007/s00421-025-05831-0, 40536521 PMC12678604

[2] Muratomi K Furuhashi Y Ushirooka N Kanno K Tanigawa S Maemura H. Metabolic efficiency in a supramaximal treadmill running test does not directly determine 400-m sprint performance. J Sports Med Phys Fitness. (2026) 66:726–31. doi: 10.23736/S0022-4707.26.17599-9, 41867032

[3] Clarke SF Murphy EF O'Sullivan O Lucey AJ Humphreys M Hogan A . Exercise and associated dietary extremes impact on gut microbial diversity. Gut. (2014) 63:1913–20. doi: 10.1136/gutjnl-2013-306541, 25021423

[4] Mancin L Paoli A Berry S Gonzalez JT Collins AJ Lizarraga MA . Standardization of gut microbiome analysis in sports. Cell Rep Med. (2024) 5:101759. doi: 10.1016/j.xcrm.2024.101759, 39368478 PMC11514603

[5] Hughes RL Holscher HD. Fueling gut microbes: a review of the interaction between diet, exercise, and the gut microbiota in athletes. Adv Nutr. (2021) 12:2190–215. doi: 10.1093/advances/nmab077, 34229348 PMC8634498

[6] Petersen LM Bautista EJ Nguyen H Hanson BM Chen L Lek SH . Community characteristics of the gut microbiomes of competitive cyclists. Microbiome. (2017) 5:98. doi: 10.1186/s40168-017-0320-4, 28797298 PMC5553673

[7] Morita H Kano C Ishii C Kagata N Ishikawa T Hirayama A . Bacteroides uniformis and its preferred substrate, α-cyclodextrin, enhance endurance exercise performance in mice and human males. Sci Adv. (2023) 9:eadd2120. doi: 10.1126/sciadv.add2120, 36696509 PMC9876546

[8] Kulecka M Fraczek B Mikula M Zeber-Lubecka N Karczmarski J Paziewska A . The composition and richness of the gut microbiota differentiate the top polish endurance athletes from sedentary controls. Gut Microbes. (2020) 11:1374–84. doi: 10.1080/19490976.2020.1758009, 32401138 PMC7524299

[9] Carlone J Rossi C Bianco A Drid P Parisi A Fasano A. Exploring the gut microbiome in combat sports: a systematic scoping review. Sports (Basel). (2026) 14:19. doi: 10.3390/sports14010019, 41590961 PMC12846275

[10] Humińska-Lisowska K Zielińska K Mieszkowski J Michałowska-Sawczyn M Cięszczyk P Łabaj PP . Microbiome features associated with performance measures in athletic and non-athletic individuals: a case-control study. PLoS One. (2024) 19:e0297858. doi: 10.1371/journal.pone.0297858, 38381714 PMC10880968

[11] Li Y Cheng M Zha Y Yang K Tong Y Wang S . Gut microbiota and inflammation patterns for specialized athletes: a multi-cohort study across different types of sports. mSystems. (2023) 8:e0025923. doi: 10.1128/msystems.00259-23, 37498086 PMC10470055

[12] Bycura D Santos AC Shiffer A Kyman S Winfree K Sutliffe J . Impact of different exercise modalities on the human gut microbiome. Sports. (2021) 9:14. doi: 10.3390/sports9020014, 33494210 PMC7909775

[13] Varghese S Rao S Khattak A Zamir F Chaari A. Physical exercise and the gut microbiome: a bidirectional relationship influencing health and performance. Nutrients. (2024) 16:3663. doi: 10.3390/nu16213663, 39519496 PMC11547208

[14] Scheiman J Luber JM Chavkin TA MacDonald T Tung A Pham LD . Meta-omics analysis of elite athletes identifies a performance-enhancing microbe that functions via lactate metabolism. Nat Med. (2019) 25:1104–9. doi: 10.1038/s41591-019-0485-4, 31235964 PMC7368972

[15] Zhang L Li H Song Z Liu Y Zhang X. Dietary strategies to improve exercise performance by modulating the gut microbiota. Foods. (2024) 13:1680. doi: 10.3390/foods13111680, 38890909 PMC11171530

[16] Chen Y Yang K Xu M Zhang Y Weng X Luo J . Dietary patterns, gut microbiota and sports performance in athletes: a narrative review. Nutrients. (2024) 16:1634. doi: 10.3390/nu16111634, 38892567 PMC11175060

[17] Garber CE Blissmer B Deschenes MR Franklin BA Lamonte MJ Lee IM . American College of Sports Medicine position stand. Quantity and quality of exercise for developing and maintaining cardiorespiratory, musculoskeletal, and neuromotor fitness in apparently healthy adults: guidance for prescribing exercise. Med Sci Sports Exerc. (2011) 43:1334–59. doi: 10.1249/MSS.0b013e318213fefb, 21694556

[18] Zhao W-H Huang Z-P Zhang X He L Willett W Wang J-L . Reproducibility and validity of a Chinese food frequency questionnaire. Biomed Environ Sci. (2010) 23:1–38. doi: 10.1016/s0895-3988(11)60014-720486429

[19] Chen S Zhou Y Chen Y Gu J. Fastp: an ultra-fast all-in-one FASTQ preprocessor. Bioinformatics. (2018) 34:i884–90. doi: 10.1093/bioinformatics/bty560, 30423086 PMC6129281

[20] Langmead B Trapnell C Pop M Salzberg SL. Ultrafast and memory-efficient alignment of short DNA sequences to the human genome. Genome Biol. (2009) 10:R25. doi: 10.1186/gb-2009-10-3-r25, 19261174 PMC2690996

[21] Li D Liu CM Luo R Sadakane K Lam TW. MEGAHIT: an ultra-fast single-node solution for large and complex metagenomics assembly via succinct de Bruijn graph. Bioinformatics. (2015) 31:1674–6. doi: 10.1093/bioinformatics/btv033, 25609793

[22] Zhu W Lomsadze A Borodovsky M. Ab initio gene identification in metagenomic sequences. Nucleic Acids Res. (2010) 38:e132. doi: 10.1093/nar/gkq275, 20403810 PMC2896542

[23] Steinegger M Söding J. MMseqs2 enables sensitive protein sequence searching for the analysis of massive data sets. Nat Biotechnol. (2017) 35:1026–8. doi: 10.1038/nbt.3988, 29035372

[24] Buchfink B Xie C Huson DH. Fast and sensitive protein alignment using DIAMOND. Nat Methods. (2015) 12:59–60. doi: 10.1038/nmeth.3176, 25402007

[25] Bolyen E Rideout JR Dillon MR Bokulich NA Abnet CC Al-Ghalith GA . Reproducible, interactive, scalable and extensible microbiome data science using QIIME 2. Nat Biotechnol. (2019) 37:852–7. doi: 10.1038/s41587-019-0209-9 31341288 PMC7015180

[26] Segata N Izard J Waldron L Gevers D Miropolsky L Garrett WS . Metagenomic biomarker discovery and explanation. Genome Biol. (2011) 12:R60. doi: 10.1186/gb-2011-12-6-r60, 21702898 PMC3218848

[27] Wu T Hu E Xu S Chen M Guo P Dai Z . ClusterProfiler 4.0: a universal enrichment tool for interpreting omics data. Innovation (N Y). (2021) 2:100141. doi: 10.1016/j.xinn.2021.100141, 34557778 PMC8454663

[28] Peng C Chen Q Tan S Shen X Jiang C. Generalized reporter score-based enrichment analysis for omics data. Brief Bioinform. (2024) 25:bbae116. doi: 10.1093/bib/bbae116, 38546324 PMC10976918

[29] Wang H Ma H Yan H Pei Z Zhao J Zhang H . Study on the effect of *Bifidobacterium adolescentis* CCFM1066 on exercise performance, gut microbiota, and its metabolites in mice. Probiotics Antimicrob Proteins. (2026) 18:54–67. doi: 10.1007/s12602-025-10493-7, 40064789

[30] Mörkl S Lackner S Müller W Gorkiewicz G Kashofer K Oberascher A . Gut microbiota and body composition in anorexia nervosa inpatients in comparison to athletes, overweight, obese, and normal weight controls. Int J Eat Disord. (2017) 50:1421–31. doi: 10.1002/eat.22801, 29131365

[31] Shalmon G Ibrahim R Israel-Elgali I Grad M Shlayem R Shapira G . Differential gut microbiome profiles in long-distance endurance cyclists and runners. Life (Basel). (2024) 14:100141. doi: 10.3390/life14121703, 39768409 PMC11677284

[32] Mach N Fuster-Botella D. Endurance exercise and gut microbiota: a review. J Sport Health Sci. (2017) 6:179–97. doi: 10.1016/j.jshs.2016.05.001, 30356594 PMC6188999

[33] Kulecka M Fraczek B Balabas A Czarnowski P Zeber-Lubecka N Zapala B . Characteristics of the gut microbiome in esports players compared with those in physical education students and professional athletes. Front Nutr. (2022) 9:1092846. doi: 10.3389/fnut.2022.1092846, 36726816 PMC9884692

[34] Wang Y Bai S Yang T Guo J Zhu X Dong Y. Impact of exercise-induced alterations on gut microbiota diversity and composition: comparing effects of different training modalities. Cell Regen. (2025) 14:28. doi: 10.1186/s13619-025-00244-y, 40601060 PMC12222581

[35] Straub D Englert T Beller A Stadelmaier J Stahl M Kilian J . Resistance training reshapes the gut microbiome in a longitudinal 8-week intervention in sedentary adults. Sports Med Open. (2026) 12:21. doi: 10.1186/s40798-026-00990-6, 41834018 PMC12989468

[36] Pereira SMS José V da Silva A Martins KVC Leite LB Forte P . Can physical exercise modify intestinal integrity and gut microbiota composition? A systematic review of in vivo studies. Biol Sport. (2025) 42:13–28. doi: 10.5114/biolsport.2025.148545, 41048220 PMC12490311

[37] Combs D Landeros K Garza K Azari H Abdelrahman M Albracht-Schulte K. Exercise intensity as a modulator of gut microbiota and host metabolic health in obesity. Gut Microbes. (2026) 18:2661415. doi: 10.1080/19490976.2026.2661415, 42010766 PMC13102036

[38] Blanco-Miguez A Galvez EJC Pasolli E De Filippis F Amend L Huang KD . Extension of the Segatella copri complex to 13 species with distinct large extrachromosomal elements and associations with host conditions. Cell Host Microbe. (2023) 31:1804–1819.e9. doi: 10.1016/j.chom.2023.09.013, 37883976 PMC10635906

[39] Peng Y Tun HM. Meet the extended Segatella copri complex. Cell Host Microbe. (2023) 31:1766–9. doi: 10.1016/j.chom.2023.10.009, 37944487

[40] Zhou C Hibberd MC Lee EM Pilgaard B Vuillemin M Kiehn E . Glycoside hydrolase-mediated glucomannan catabolism in Segatella copri, a target of microbiota-directed foods for malnourished children. Proc Natl Acad Sci USA. (2025) 122:e2521522122. doi: 10.1073/pnas.2521522122, 41329729 PMC12704710

[41] Marcobal AM McConnell BR Drexler RA Ng KM Maldonado-Gomez MX Conner AMS. Highly soluble beta-glucan fiber modulates mechanisms of blood glucose regulation and intestinal permeability. Nutrients. (2024) 16:2240. doi: 10.3390/nu16142240 39064683 PMC11279855

[42] Zhang XY Chen J Yi K Peng L Xie J Gou X . Phlorizin ameliorates obesity-associated endotoxemia and insulin resistance in high-fat diet-fed mice by targeting the gut microbiota and intestinal barrier integrity. Gut Microbes. (2020) 12:1–18. doi: 10.1080/19490976.2020.1842990, 33222603 PMC7714487

[43] Li B Wang H Wang M Liang H Hu T Yang J . Genome analysis of *Bifidobacterium adolescentis* and investigation of its effects on inflammation and intestinal barrier function. Front Microbiol. (2024) 15:1496280. doi: 10.3389/fmicb.2024.1496280, 39911710 PMC11794259

[44] Lamprecht M Bogner S Schippinger G Steinbauer K Fankhauser F Hallstroem S . Probiotic supplementation affects markers of intestinal barrier, oxidation, and inflammation in trained men; a randomized, double-blinded, placebo-controlled trial. J Int Soc Sports Nutr. (2012) 9:45. doi: 10.1186/1550-2783-9-45, 22992437 PMC3465223

[45] Van Haute MJ Chacón-Vargas K Christensen CM Yumul SRP Abdulaali FF Benson AK . Comparative genomic and phenotypic analysis of potential beneficial properties of *Bifidobacterium adolescentis*. mSphere. (2025) 10:e0067325. doi: 10.1128/msphere.00673-25, 41186414 PMC12646000

[46] Yu X Tian G Wang Y Li L Huang L Zhao Y . *Bifidobacterium adolescentis* CCFM1447 effectively alleviates osteoporosis by enriching intestinal flora capable of vitamin D conversion. Front Nutr. (2025) 12:1647671. doi: 10.3389/fnut.2025.1647671, 41064290 PMC12502076

[47] Royo F Tames H Bordanaba-Florit G Cabrera D Azparren-Angulo M Garcia-Vallicrosa C . Orally administered *Bifidobacterium adolescentis* diminishes serum glutamate concentration in mice. Microbiol Spectrum. (2023) 11:e0506322. doi: 10.1128/spectrum.05063-22, 37347184 PMC10433951

